# Ginger Oleoresin Alleviated *γ*-Ray Irradiation-Induced Reactive Oxygen Species via the Nrf2 Protective Response in Human Mesenchymal Stem Cells

**DOI:** 10.1155/2017/1480294

**Published:** 2017-10-18

**Authors:** Kaihua Ji, Lianying Fang, Hui Zhao, Qing Li, Yang Shi, Chang Xu, Yan Wang, Liqing Du, Jinhan Wang, Qiang Liu

**Affiliations:** ^1^Tianjin Key Laboratory of Radiation Medicine and Molecular Nuclear Medicine, Department of Radiobiology, Institute of Radiation Medicine of Chinese Academy of Medical Science, Tianjin 300192, China; ^2^Tianjin Key Laboratory of Food and Biotechnology, School of Biotechnology and Food Science, Tianjin University of Commerce, Tianjin 300134, China; ^3^Tsingdao Lihe Exact Science & Technology Co. Ltd., Tsingdao 266111, China

## Abstract

Unplanned exposure to radiation can cause side effects on high-risk individuals; meanwhile, radiotherapies can also cause injury on normal cells and tissues surrounding the tumor. Besides the direct radiation damage, most of the ionizing radiation- (IR-) induced injuries were caused by generation of reactive oxygen species (ROS). Human mesenchymal stem cells (hMSCs), which possess self-renew and multilineage differentiation capabilities, are a critical population of cells to participate in the regeneration of IR-damaged tissues. Therefore, it is imperative to search effective radioprotectors for hMSCs. This study was to demonstrate whether natural source ginger oleoresin would mitigate IR-induced injuries in human mesenchymal stem cells (hMSCs). We demonstrated that ginger oleoresin could significantly reduce IR-induced cytotoxicity, ROS generation, and DNA strand breaks. In addition, the ROS-scavenging mechanism of ginger oleoresin was also investigated. The results showed that ginger oleoresin could induce the translocation of Nrf2 to cell nucleus and activate the expression of cytoprotective genes encoding for HO-1 and NQO-1. It suggests that ginger oleoresin has a potential role of being an effective antioxidant and radioprotective agent.

## 1. Introduction

Radiation from natural or artificial sources is a common phenomenon in our daily life [1]. However, abnormal exposure to radiation can cause side effects on individuals who are involved in nuclear mishaps, attack cleanup crews, astronauts, nuclear power plant workers, and some medical professionals whom could be professionally or accidentally exposed to radiation [2]. Furthermore, radiation therapy used as one of the most important therapy strategies for human malignancy can also injure the normal cells and tissues surrounding the tumor [3]. It represents a cause of treatment toxicity and a limiting factor for dose, volume, and technique of radiation therapy.

Human mesenchymal stem cells (hMSCs) which reside in the mesenchymal stroma are an important population of cells [4]. hMSCs were first isolated from bone marrow and could be found in almost all human organs and tissues such as kidney, vascular tissue, adipose tissue, skin, umbilical cord, and placenta [5–7]. These cells possess stem cell-like characteristics including self-renewal and multilineage differentiation into mesenchymal and nonmesenchymal lineages [8]. hMSCs have been proved to participate in the regeneration of ionizing radiation-damaged tissues. However, hMSCs themselves can also be damaged by ionizing radiation [9]. When irradiated in vitro with increasing doses, the human bone-derived MSC was reported with the phenomenon of greatly reduced self-renewal, proliferation, and differentiation capabilities [10]. Thus, there is an urgent need for exploring natural effective radioprotectors which could be used to protect normal cells, especially hMSCs when exposed to radiation.

Radiation-induced damages are mediated directly by DNA single-strand breaks, DNA double-strands breaks, and chromosome damage and indirectly due to the production of reactive oxygen species (ROS) [1, 11–13]. ROS makes a large part of contributions to radiation-induced damages, so much of the efforts in the past were made in exploring potential natural antioxidants without obvious side effects to ameliorate radiation-induced toxicities [14]. Many naturally occurring phenolic compounds, such as bioactive substances in plants, grape seed proanthocyanidins, tea polyphenol, curcumin, and ginger oleoresin, have been reported to possess antioxidant properties [15–20]. Ginger (*Zingiber officinale* Roscoe, Zingiberaceae) is one of the most widely consumed spice and condiment for foods and beverages [21]. It has also been used as a remedy for common cold, motion sickness, nausea, digestive disorders, rheumatism, indigestion, and osteoarthritis for a long time in traditional oriental medicine [22]. Ginger oleoresin is a complex mixture which is extracted from Zingiberaceae and is rich in gingerols and shogaols. [6]-Gingerol, [6]-shogaols, and their derivatives are considered as chemopreventive candidates against ROS stress and cancer due to their property of activating the Nrf2-ARE signaling pathway in different types of human cells [23–26]. Furthermore, many researches have reported that [6]-gingerol can prevent UVB or gamma radiation-induced cell damage both in vitro and in vivo [27, 28]. Nrf2, which is regarded as a redox-sensitive prosurvival transcription factor, is maintained at a very low level through Keap1-mediated ubiquitylation and subsequent proteasome-mediated degradation. However, when cells are exposed to oxidative situation, the level of Nrf2 rises, meanwhile Nrf2 translocates into the cell nucleus and binds to the antioxidant response element (ARE) located in the promoter region of cytoprotective genes and upregulates their transcription [29]. The Nrf2 target genes can encode proteins with diverse cellular functions including intracellular redox-balancing proteins such as heme oxygenase-1 (HO-1) and glutamate-cysteine ligase that eliminate ROS and maintain the cellular redox capacity and phase II detoxifying enzymes such as NAD(P)H:quinone oxidoreductase 1 (NQO-1) [29].

Ginger oleoresin, which is extracted from ginger by a supercritical CO_2_ fluid-extracted method, is a nonvolatile pungent mixture [30]. Ginger oleoresin is mainly composed of gingerols and shogaols [30]. Therefore, we hypothesized that ginger oleoresin may be a potent radioprotective agent in hMSCs cells via Nrf2 protective response. In the present study, we evaluated that ginger oleoresin can protect hMSCs against radiation-inducing cell damage. Based on these findings, we further verified the radioprotective molecular mechanism of ginger oleoresin focusing on the roles of Nrf2 and its target antioxidant enzymes.

## 2. Materials and Methods

### 2.1. Cell Culture

Human mesenchymal stem cells (hMSCs) were obtained from the National Engineering Research Center (NERC) of China, cultured in Dulbecco's modified Eagle's medium-F12 (Hyclone, Logan, UT, USA) containing 10% fetal bovine serum (Gibco, Carlsbad, CA, USA) and antibiotics (100 U/mL penicillin and 100 *μ*g/mL streptomycin) (Invitrogen, Carlsbad, CA, USA) in an atmosphere with 5% CO_2_ at 37°C and passaged every two days.

### 2.2. Treatment with Ginger Oleoresin and Irradiation Schedule

Ginger oleoresin was presented by Tianjin University of Commerce and diluted in the equal volume of dimethyl sulfoxide (DMSO) (Dingguo, BJ, China) to prepare the stock solution. Then, the stock solution was further diluted to the required concentration using cell culture medium. For cytotoxicity determination of ginger oleoresin, hMSCs were exposed to 10^−3^, 10^−4^, 10^−5^, and 10^−6^ g/mL ginger oleoresin and cultured for 24 h, 48 h, and 72 h. For radioprotective effect determination, hMSCs were pretreated with 10^−4^ g/mL ginger oleoresin and cultured for 2 h followed by exposure to *γ*-rays at the Irradiation Center. After irradiation, the cells were subsequently incubated for 24 h. ^137^Cs was used as the irradiation source (AECL, Canada). hMSCs cells (±ginger oleoresin in medium) were irradiated at a dose of 4 Gy. DMSO was used as a vehicle control. hMSCs + ginger oleoresin without *γ*-ray irradiation were also studied.

### 2.3. Cell Viability Assay

To detect cell viability, the 3-(4,5-dimethylthia-zol-2-yl)-2,5-diphenyltetrazolium bromide (MTT) (Solarbio, Beijing, China) assay was performed [31]. MTT can be reduced to purple formazan in the mitochondria of living cells. The absorbance of formazan solution can be quantified by a spectrophotometer, and hence, this method can be applied to assess the cytoprotective ability and the toxicity of ginger oleoresin based on the viability of cells. For toxicity test, hMSCs cells (5 × 10^3^ cells/well) were seeded in a 96-well plate and treated with the indicated dose of ginger oleoresin for the indicated time period. For cytoprotective determination, hMSCs were seeded in a 96-well plate and pretreated with 10^−4^ g/mL ginger oleoresin or the same volume of DMSO and cultured for 2 h before exposure to 4 Gy *γ*-rays. Then, the hMSCs cells were cultured for 24 h and added 10 *μ*L MTT (5 mg/mL) solution. The cells were incubated at 30°C for 4 h. The supernatants were discarded, and then 150 *μ*L DMSO was added. The cell viability was determined by measuring the absorbance at 492 nm on a multifunctional microplate reader (BioTek, Winooski, VT).

### 2.4. Intracellular ROS Measurements

Measurements of intracellular ROS levels were performed using the 2′,7′-dichlorodihydrofluoroscein diacetate (DCFH_2_-DA) (Sigma, USA) method [32]. DCFH_2_-DA is able to diffuse through the cell membrane and be hydrolysed by intracellular esterases to produce DCFH_2_. The nonfluorescent DCFH_2_ is oxidized by intracellular ROS and results in fluorescent DCF. The treated cell samples (hMSCs, hMSCs + ginger oleoresin, hMSCs + *γ*-ray irradiation, and hMSCs + ginger oleoresin + *γ*-ray irradiation) were incubated in the presence of 10 *μ*M DCFH_2_-DA in DMEM-F12 medium at 37°C for 20 min and then washed three times with DMEM-F12 medium to remove the extracellular DCFH_2_-DA. The fluorescence values of DCF inside the cells were monitored to evaluate and detect intracellular ROS by excitation at 498 nm and emission 530 nm using a flow cytometer (BriCyte E6, Mindray, Shenzhen, China) and inverted fluorescence microscope (Leica DMI3000B, German).

### 2.5. Comet Assay

The DNA damage in an individual cell was measured by using the gel electrophoresis-based comet assay [33]. Under alkaline conditions, the negatively charged DNA supercoils with broken ends were able to migrate toward the anode during electrophoresis. But the DNA supercoils without breaks prevented migration. For comet assay, hMSCs were pretreated with 10^−4^ g/mL ginger oleoresin and cultured for 2 h followed by exposure to *γ*-rays at the Irradiation Center. After irradiation, the cells were subsequently incubated for 24 h. The comet assay was performed as described before [33]. Briefly, the treated cell samples were applied to prepare single-cell suspensions using trypsin disaggregation. The single-cell suspensions (30 *μ*L) were mixed with low melting point agarose gel (70 *μ*L) (Promega, Madison, WI). The mixture drop was added to a slide with agarose gel (Biowest, Nuaille, France) and then was lysed. Subsequently, the cells were exposed to alkali for DNA unwinding and electrophoresis. After electrophoresis, the slides were neutralized, stained with ethidium bromide (EB), and observed using a fluorescence microscope (ETLPSE 90i, Nikon, Japan). The result images were analyzed using CASP software.

### 2.6. Quantitative Real-Time PCR

After treatment with or without ginger oleoresin and *γ*-ray irradiation, cells were cultured for 24 h. RNA extraction, cDNA synthesis, and quantitative real-time PCR were performed as described before [34]. The total RNA in each group was isolated using Trizol reagent (Invitrogen, Carlsbad, CA) following the manufacturer's protocol, and equal amounts of RNA were reverse transcribed to cDNA using a PrimeScript RT reagent kit (Takara, Dalian, China). *Nrf2*, *HO-1*, *NQO-1*, and *GAPDH* mRNA transcription levels were determined using Fast Start Universal SYBR Green Master (Roche, Indianapolis, IN). The sequences of primers used in this study are listed in Table 1. The quantitative real-time PCR data presented are relative mRNA levels normalized to GAPDH, and the value from the untreated control group was set as 1.

### 2.7. Immunoblotting Analysis

After cells were treated as described above and incubated for 24 h, the expression levels of Nrf2, HO-1, NQO-1, TBP, and TUBLIN were detected using immunoblotting assay. Briefly, cells were lysed with RIPA protein extraction reagent (Bestbio, Shanghai, China) to extract the total protein. The protein concentrations of the extracts were measured by using a bicinchoninic acid kit (Beyotime, Beijing, China) according to the manufacturer's instructions. Equal amounts of extracts were fractionated by SDS-PAGE, and then they were transferred to nitrocellulose membranes (Millipore, Massachusetts, USA). After being transferred, the proteins on the nitrocellulose membranes were blotted with the antibodies indicated. Nrf2, HO-1, NQO-1, TBP, and TUBLIN primary antibodies were purchased from Abcam (Abcam, Cambridge, UK). The intensities of protein bands were measured using Quantity One software (Bio-rad, Hercules, CA).

### 2.8. Determination of Nuclear Levels of Nrf2

Nuclear levels of Nrf2 were determined by immunofluorescence assay and immunoblotting assay. For immunofluorescence assay, the cells were fixed with 4% paraformaldehyde; and the cell membranes were disrupted using 0.3% TrintonX-100 after the cells were treated and incubated. Subsequently, the cells were labelled with Nrf2 antibody and secondary antibody IgG. Then, the cells were stained with DAPI for nuclear staining. The stained cells were examined using an EVOS inverted fluorescence microscope (Thermo Fisher, MA, USA). Overlay images were recorded by superimposing simultaneous images from two different channels. For immunoblotting assay, the proteins in the cytoplasm and nucleus were isolated using NE-PER Nuclear and Cytoplasmic extraction reagent (Thermo Scientific, Waltham, USA).

### 2.9. siRNA Knockdown Studies

Nrf2 siRNA was used for knocking down Nrf2 in hMSCs to explore whether the antioxidant effect of ginger oleoresin was through the Nrf2 pathways. RNA interference assay was performed as described before [35]. Briefly, 7 × 10^4^ cells were inoculated into a 6-well plate and 1.8 mL fresh medium was replaced for every well. Then, 2 nmol siRNA (GenePharma, Suzhou, China) mixed with 5 *μ*L Lipofectamine 2000 RNAiMAX Reagent (Thermo Scientific, Waltham, USA) and 193 *μ*L Opti-MEM reduced serum culture medium (Thermo Scientific, Waltham, USA) were added into each well. Cells were further cultured for 1~3 days for transfection and gene knockdown. Knockdown hMSCs were treated with or without ginger oleoresin for 2 h followed by a radiation dose of 4 Gy and incubated for 24 h. MTT assay was used to detect whether the radiation protection effect of ginger oleoresin was lost in Nrf2-silenced hMSCs. Cells were further subjected to RNA extraction for quantitative real-time PCR assay and protein extraction for Western blot assay.

### 2.10. Statistical Analysis

Data are represented as means ± standard deviations. Statistical analysis was performed using SPSS 19.0 software. Student's *t*-test was performed for the analysis of differences between the two groups. *p* < 0.05 was indicated as statistically significant.

## 3. Results

### 3.1. Toxicity of Ginger Oleoresin to Human Mesenchymal Stem Cells (hMSCs)

**Figure 1:**
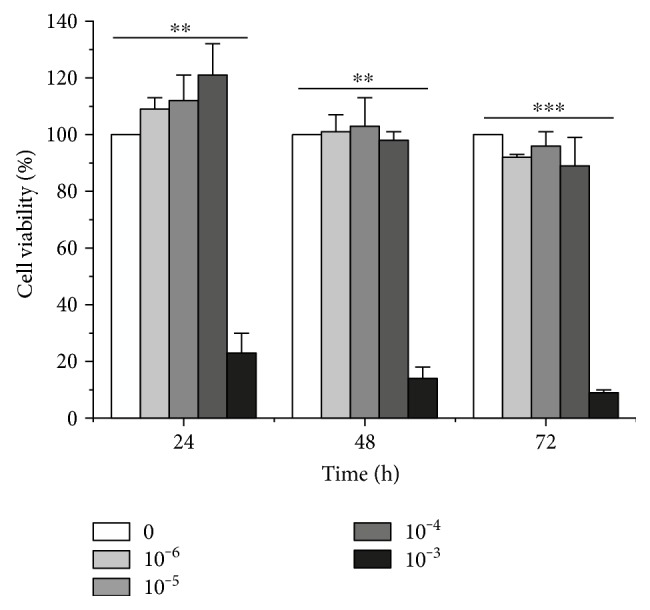
Cytotoxicity of ginger oleoresin in human mesenchymal stem cells (hMSCs) for 24, 36, and 48 h. Cell viability was measured using the MTT assay. The results are the mean ± SD of three experiments, each in triplicate. ^∗∗^*p* < 0.01 10^−3^ g/mL treated versus control. ^∗∗∗^*p* < 0.001 10^−3^ g/mL treated versus control.

We firstly used the MTT method to evaluate the toxicity of ginger oleoresin to human mesenchymal stem cells (hMSCs) and to determine the treatment doses. As shown in Figure 1, when cells were treated with ginger oleoresin for 24 h, 48 h, and 72 h, there was no significant cell toxicity below 10^−4^ g/mL, but 10^−3^ g/mL was toxic. Therefore, the concentration of 10^−4^ g/mL was chosen for the subsequent bioassays.

### 3.2. Ginger Oleoresin Significantly Protected Human Mesenchymal Stem Cells against Radiation-Induced Cytotoxicity

**Figure 2:**
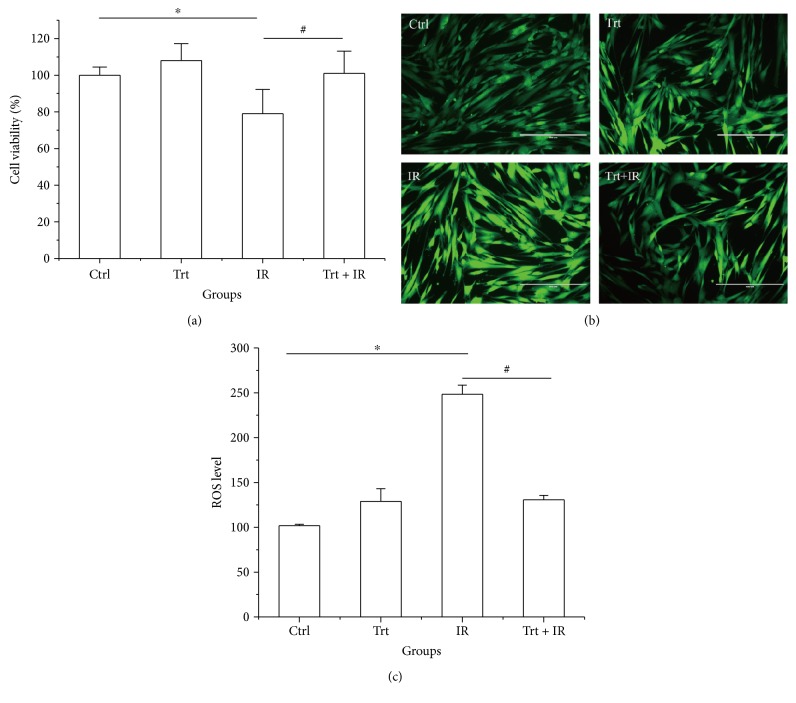
Ginger oleoresin increased the cell viability and reduced the *γ*-ray irradiation-induced ROS level in hMSCs. Cell viability was measured using the MTT assay (a). The level of ROS was measured using DCF fluorescence with a fluorescence microscope (b) and flow cytometry (c). Cells were untreated (Ctrl) or treated with ginger oleoresin (10^−4^ g/mL) (Trt) and *γ*-ray irradiation (4 Gy) (IR) or pretreated with ginger oleoresin (10^−4^ g/mL) and then *γ*-ray irradiation (4 Gy) (Trt + IR). Results are expressed as mean ± SD (*n* = 4). ^∗^*p* < 0.05 IR versus untreated control (Ctrl). ^#^*p* < 0.05 Trt + IR versus IR.

**Figure 3:**
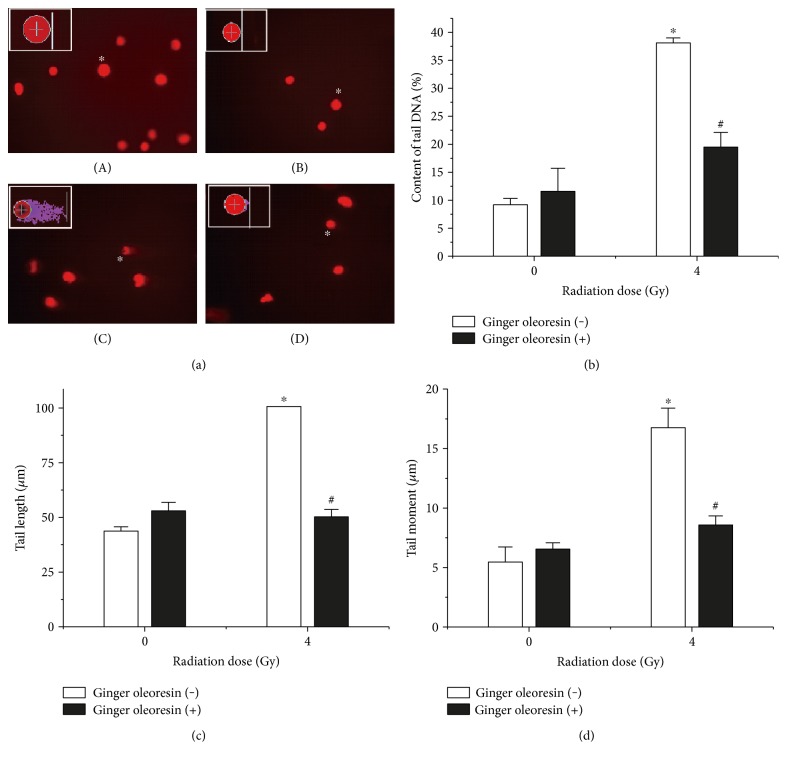
Ginger oleoresin reduced the IR-induced DNA strand breaks in hMSCs. The levels of DNA strand breaks were assayed by comet assay. (a) Representative micrographs—(A): untreated group; (B): treated with ginger oleoresin only; (C): treated with IR only; and (D): pretreated with ginger oleoresin and then with IR. (b) Tail DNA%. (c) Tail length. (d) Tail moment. Results are expressed as mean ± SD (*n* = 4). ^∗^*p* < 0.05 IR versus untreated control (Ctrl). ^#^*p* < 0.05 ginger oleoresin + IR versus IR.

The protection conferred by ginger oleoresin against radiation-induced cytotoxicity was evaluated. Human mesenchymal stem cells (hMSCs) were pretreated with 10^−4^ g/mL ginger oleoresin for 2 h before *γ*-ray irradiation. As shown in Figure 2(a), we demonstrated that the pretreatment of hMSCs cells with ginger oleoresin before radiation significantly increased cell survival rates as compared to cells treated with radiation alone. To our knowledge, the homeostasis of cellular redox status is very important for maintaining the normal functions of cells [36], and ionizing radiation is known to modulate the cellular redox status via inducing the production of reactive oxygen species [1]. The effect of ginger oleoresin to modulate cellular redox status was then evaluated by monitoring changes in ROS levels using DCFH-DA assay. As observed in a fluorescence microscope (Figure 2(b)) and measured in flow cytometry (Figure 2(c)), treatment with 4 Gy *γ*-ray irradiation alone significantly increased the intracellular level of ROS (248.40 ± 10.13) compared to the untreated control group (101.70 ± 1.72), which could be reverted by pretreatment with 10^−4^ (g/mL) ginger oleoresin (130.70 ± 4.99), while this dose of ginger oleoresin alone had no obvious effect on ROS levels (129.00 ± 13.96). These results indicated that ginger oleoresin is able to enhance intracellular redox capacity and inhibit *γ*-ray irradiation-induced oxidative stress. In addition, ionizing radiation is very harmful to cells through inducing widespread biomolecule damages, such as DNA, protein, and lipid damages [37], among which DNA damage is the most important target of IR. Protection of ginger oleoresin to DNA damage was investigated via comet assay. As shown in Figure 3(), the radiated human mesenchymal stem cells (hMSCs) resulted in an increase in the levels of all comet parameters (tail DNA%, tail length, and tail moment), whereas pretreatment of ginger oleoresin before radiation inhibited the increase of these parameters significantly. These results indicated the protective effect of ginger oleoresin on IR-induced DNA damage.

### 3.3. Ginger Oleoresin Induced the Enhancement of Antioxidant Pathway in Human Mesenchymal Stem Cells (hMSCs)

**Figure 4:**
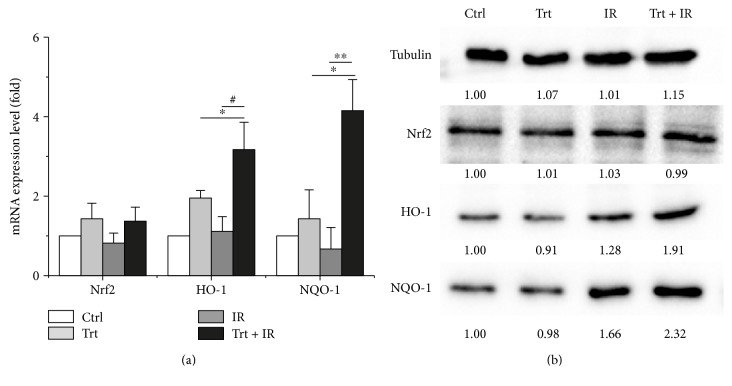
Expression properties of Nrf2 and its downstream genes encoding for HO-1 and NQO-1 in hMSCs when untreated (Ctrl) or treated with ginger oleoresin alone (Trt) and ionizing radiation alone (IR) or pretreated with ginger oleoresin and then ionizing radiation (Trt + IR). (a) mRNA levels. Results are expressed as mean ± SD (*n* = 3). ^∗^*p* < 0.05 Trt + IR versus Trt. ^#^*p* < 0.05 Trt + IR versus IR. ^∗∗^*p* < 0.01 Trt + IR versus IR. (b) Protein levels.

Kelch-like ECH-associated protein 1- (Keap1-) nuclear factor erythroid 2-related factor 2- (Nrf2-) antioxidant response element (ARE) pathway is reported as one of the most important defense mechanisms against oxidative stress [38]. Then, we tested the ability of ginger oleoresin to induce Nrf2 and downstream genes encoding for heme oxygenase-1 (HO-1) and NAD(P)H:quinone oxidoreductase 1(NQO-1) in mRNA level via qRT-PCR and protein level via Western blotting. As shown in Figure 4(a), ginger oleoresin enhanced the transcription of Nrf2 very weakly, and the results were not statistically significant (*p* > 0.05). However, the mRNA levels of HO-1 and NQO-1 were significantly induced by ginger oleoresin, especially for the group treated with ginger oleoresin and IR. And the variation pattern of Nrf2, HO-1, and NQO-1 at protein levels was consistent with that of mRNA levels (Figure 4(b)). These data indicated that ginger oleoresin induced the enhancement of the antioxidant pathway in mesenchymal stem cells not by activating the expression of Nrf2.

### 3.4. Knocking Down Nrf2 Significantly Abrogated the Ginger Oleoresin Radiation Protective Effect and Induced Antioxidant Pathway in hMSCs

**Figure 5:**
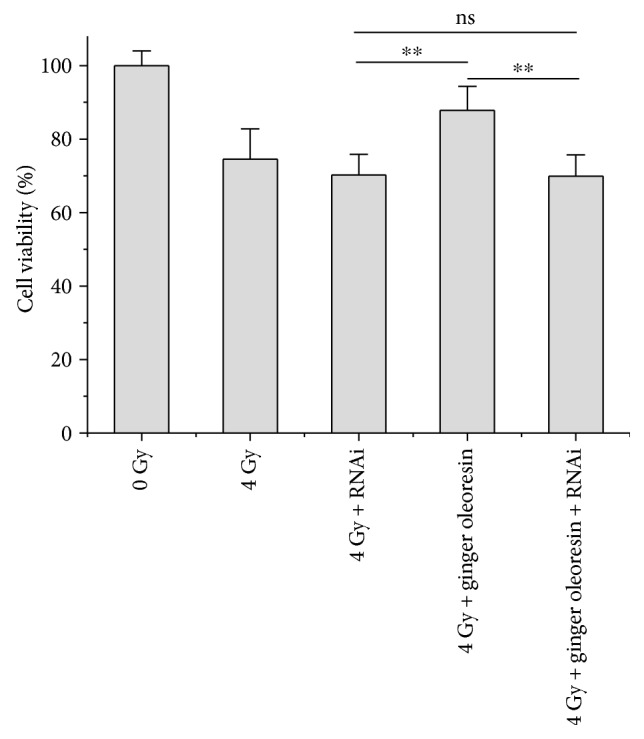
Knocking down Nrf2 reversed the radiation protective effect of ginger oleoresin on hMSCs. Results are expressed as mean ± SD (*n* = 5). ^∗∗^*p* < 0.01.

**Figure 6:**
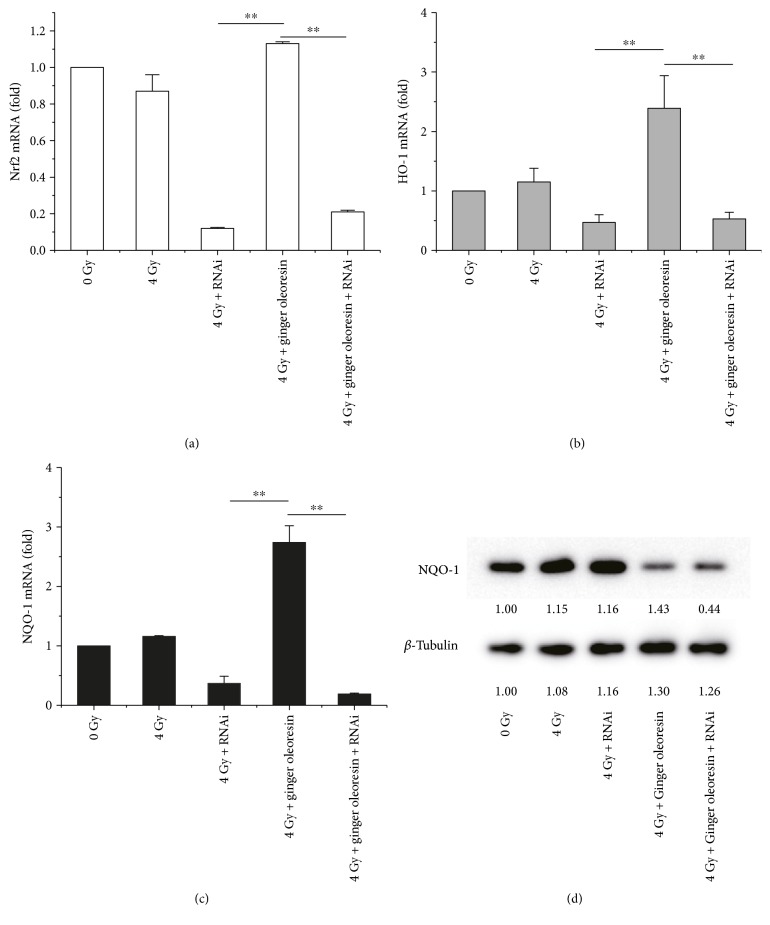
Knocking down Nrf2 reversed the activation of HO-1 and NQO-1 offered by ginger oleoresin in hMSCs when cotreated with ionizing radiation. (a) mRNA levels of Nrf2. (b) mRNA levels of HO-1. (c) mRNA levels of NQO-1. (d) Protein levels of NQO-1. Results are expressed as mean ± SD (*n* = 3). ^∗∗^*p* < 0.01.

To further test the role of Nrf2 in the radiation protective effect of ginger oleoresin on hMSCs, Nrf2 knockdown assay was carried out. hMSCs were transfected with Nrf2 siRNA or scrambled siRNA to create a knockdown or negative control. After 3 days, transfected cells with ablated Nrf2 were treated as described above (with or without ginger oleoresin and with or without irradiation) and were further monitored for the cell viability, transcription, and expression of Nrf2, HO-1, and NQO-1 by MTT, qRT-PCR, and immunoblotting, respectively. When pretreated with ginger oleoresin and then irradiation, the cell viability of hMSCs was significantly higher than that treated with irradiation alone (Figure 5) which was consistent with the abovementioned Figure 2(a). The result suggested that ginger oleoresin has a protective effect on hMSCs. However, the radiation protective effect of ginger oleoresin on hMSCs was lost when Nrf2 was knocked down (Figure 5). In addition, as shown in Figures 6(a), 6(b), and 6(c), when pretreated with ginger oleoresin and then irradiation, the mRNA levels of HO-1 and NQO-1 genes were significantly increased compared to those treated with irradiation alone. Interestingly, knocking down the Nrf2 pathway significantly abrogated the transcription of HO-1 and NQO-1 genes. Further, reduction of NQO-1 expression was also observed when the Nrf2 gene was knocked down (Figure 6(d)). These results confirmed that Nrf2 functions as a central regulator for HO-1 and NQO-1 activation. All these data illuminated that ginger oleoresin may play a radiation protective effect on hMSCs through Nrf2 and its induced antioxidant pathway.

### 3.5. Ginger Oleoresin Mediated the Nuclear Translocation of Nrf2

**Figure 7:**
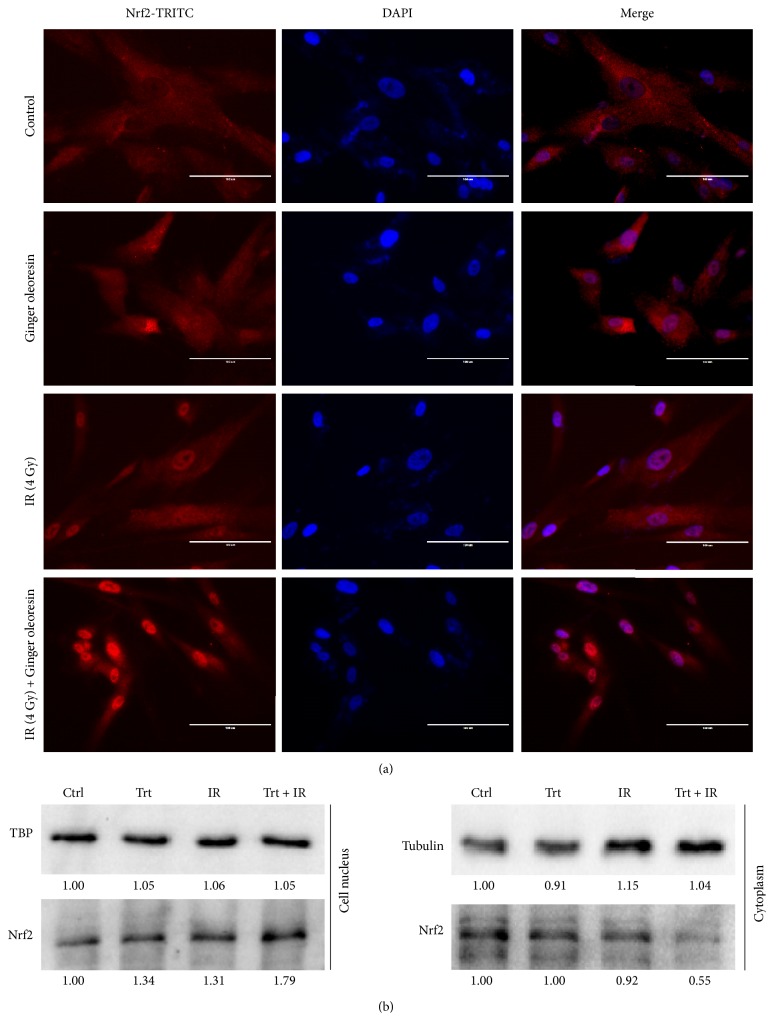
Ginger oleoresin induced nucleus translocation of Nrf2 in hMSCs when treated with ionizing radiation. (a) hMSCs when not treated or treated with ginger oleoresin alone or treated with ionizing radiation alone or pretreated with ginger oleoresin and then with ionizing radiation were stained with TRITC-labelled anti-Nrf2 antibody and DAPI. Nrf2-TRITC (left), DAPI (mid), merge (right) are shown. (b) Distribution of Nrf2 in the cytoplasm or nucleus in hMSCs. When pretreated with ginger oleoresin and then ionizing radiation, Nrf2 in the nucleus was increased.

Many reports have suggested that Nrf2 is stabilized, increased, and translocated into the nucleus under oxidation conditions [29]. Because of the central role of the Nrf2 pathway to regulate the expression of HO-1 and NQO-1, we hypothesized that ginger oleoresin might promote the nuclear translocation of Nrf2 in hMSCs. To test this, we analyzed the distribution of Nrf2 in the cytoplasm and the nucleus of hMSCs using immunofluorescence assay. As shown in Figure 7(a), when cells were treated with or without ginger oleoresin alone or combined with irradiation, the red fluorescence of TRIC-Nrf2 cannot form a clear nucleus structure. When merged with blue fluorescence of a DAPI-stained nucleus, the red fluorescence was not observed in the cell nucleus. When cells were pretreated with ginger oleoresin and irradiated with *γ*-ray radiation, the TRIC-Nrf2 red fluorescence formed a clear nucleus structure located on the blue fluorescence of the DAPI-stained nucleus (Figure 7(a)). To further demonstrate the nuclear translocation of Nrf2, we extracted the cytoplasm proteins and the nucleus protein of cells which were treated with *γ*-ray irradiation alone or pretreated with ginger oleoresin then *γ*-ray irradiation, respectively. Subsequently, the Nrf2 of the cytoplasm and nucleus was detected using immunoblotting. Consistent with the immunofluorescence results, when pretreated with ginger oleoresin, the Nrf2 levels in the cell nucleus increased (Figure 7(b)). These results suggested that ginger oleoresin induced the nucleus translocation of Nrf2.

## 4. Discussion

In the current study, we show that ginger oleoresin treatment prevents IR-induced cell injury and reduces IR-induced ROS generation in hMSCs (Figure 2). Radiation exposure from medical diagnosis, cancer therapy, nuclear exposure, and spaces flight is a kind of physical stress that increases the oxidative pressure and leads to the disturbance of cellular redox homeostasis through increasing the production of reactive oxygen species [39]. High levels of ROS can result in further oxidative damage of DNA, lipids, and protein and induce the activation of apoptotic pathway [40], so maintenance of cellular redox homeostasis is important to maintain cell viability and normal physiological responses [14]. There were studies showing that the natural source of ginger oleoresin could protect against oxidative pressure and damages. [6]-Gingerol, a major constituent of ginger oleoresin, has been found to possess many diverse pharmacologic effects including antioxidant, anti-inflammatory, and anticancer activities [23, 40, 41]. Besides, [6]-shogaol, another component of ginger oleoresin, was suggested to exhibit the most potent of antioxidant and anti-inflammatory properties in RAW 264.7 cells, although its content was much lower than gingerols [23, 42]. On the basis of the above research, it thus seems receivable that the hMSCs cell protection effects against IR by ginger oleoresin are due to the suppression of ROS production induced by IR.

Additionally, it is well known that DNA is one of the major targets of ROS. In our current study, ginger oleoresin was proved to be able to protect hMSCs against IR-induced DNA double-strand breaks. The result from comet assay demonstrates that treatment of hMSCs by ginger oleoresin can greatly reduce radiation-induced DNA damages (Figure 3). However, the molecular mechanism of ginger oleoresin on ROS scavenging in hMSCs remains unknown. Many investigators have shown that redox-sensitive prosurvival transcription factor Nrf2 plays a primary role in scavenging the ROS [38, 43]. Consequently, the Nrf2 signaling pathway is firstly taken into account for the ROS-scavenging ability of ginger oleoresin. In the present study, the cytoprotective genes encoding for HO-1 and NQO-1 were induced on both mRNA and protein levels when pretreated with ginger oleoresin and then ionizing radiation; however, the expression of the key regulatory factor Nrf2 was observed to be not obviously changed (Figure 4). To further investigate the role of Nrf2 on the radiation protective effect on hMSCs and the induction of HO-1 and NQO-1, siRNA knocking down of Nrf2 was introduced. Our data highlighted that the radiation protective effect was lost (Figure 5), and the expression of HO-1 and NQO-1 was significantly decreased when Nrf2 was knocking down (Figure 6), confirming the critical role of Nrf2 on radiation protective effect on hMSCs and regulating the downstream gene expression. As the Nrf2 expression was not responsible for its regulatory function, we hypothesized that the activation of Nrf2 may be through its translocation into the cell nucleus. On the basis of this consideration, the distribution and positioning of Nrf2 were analyzed by immunofluorescence and immunoblotting. Our data clearly showed that the ginger oleoresin induced the translocation of Nrf2 into the cell nucleus to activate the Nrf2 signaling pathway (Figure 7). Interestingly, we also observed that the nuclear levels of Nrf2 in hMSCs were not increased when cells were treated with ginger oleoresin alone but significantly increased when cells were treated with ginger oleoresin and radiation. Nrf2 has been proved to be maintained at a low level through Keap1-mediated ubiquitylation and 26S proteasome-mediated degradation. However, Nrf2 is stabilized, and Nrf2 levels rise when cells are exposed to electrophiles or oxidants [29]. In our study, it is perhaps that Nrf2 was bounded by Keap1 and could not translocate into the cell nucleus by ginger oleoresin when treated with ginger oleoresin alone. Nevertheless, Nrf2 was released and stabilized when exposed to irradiation. Then, the stabilized Nrf2 can be translocated into the cell nucleus by ginger oleoresin. These results suggested that the ginger oleoresin could alleviate *γ*-ray irradiation-induced reactive oxygen species (ROS) via the Nrf2 protective response in human mesenchymal stem cells (hMSCs).

In line with our experimental finding, [6]-gingerol has been reported to protect against UVB-induced ROS production and COX-2 expression in vitro and vivo [27]. Meanwhile, ginger essential oil and [6]-shogaol have recently been certified to protect against *γ*-ray irradiation-induced oxidative stress, clastogenic damage, and intestinal mucosa injury in mice [44, 45]. Compared to the above studies, our data extend previous findings and further confirm the protective effect of ginger oleoresin on hMSCs under IR conditions. Given the important role of hMSCs in the repair of radiation-induced tissue damage, our current study has a more important significance for radiation protection. Besides, we further investigated the molecular mechanism of ginger oleoresin to reduce IR-induced ROS generation, which is found to be regulated through the translocation of Nrf2.

In summary, ginger oleoresin pretreatment prevents IR-induced cell injury in hMSCs via reducing IR-induced ROS production, which are related to the nuclear translocation of Nrf2 and its regulation of downstream gene expression of HO-1 and NQO-1. Our study paves the way for prospective drug development of ginger oleoresin as natural resources against IR-induced injury. However, it should be noted that the scavenging ability of ginger oleoresin should be further elucidated *in vivo*.

## Figures and Tables

**Table 1:** List of primers used for QRT-PCR.

Gene	Sequence (5′-3′)
Nrf2	(Forward) TCAGCGACGGAAAGAGTATGA
	(Reverse) CCACTGGTTTCTGACTGGATGT
HO-1	(Forward) AGAGGGAATTCTCTTGGCTGGCTT
	(Reverse) ATGCCATAGGCTCCTTCCTCCTTT
NQO-1	(Forward) AGGAAGAGCTAATAAATCTCTTCTTTGCTG
	(Reverse) TCATATTGCAGATGTACGGTGTGGATTTAT
GADPH	(Forward) TGACTTCAACAGCGACACCCA
	(Reverse) CACCCTGTTGCTGTAGCCAAA
